# *BRAF* V600E mutation and the Bethesda System for Reporting Thyroid Cytopathology of fine-needle aspiration biopsy for distinguishing benign from malignant thyroid nodules

**DOI:** 10.1097/MD.0000000000027167

**Published:** 2021-09-17

**Authors:** Danli Sheng, Xiaoli Yu, Hui Li, Murui Zhang, Jianzhong Chen

**Affiliations:** a Department of Pathology, Sir Run Run Shaw Hospital, School of Medicine, Zhejiang University, Hangzhou, China; b Institute of Immunology, School of Medicine, Zhejiang University, Hangzhou, China.

**Keywords:** *BRAF* V600E, fine-needle aspiration, papillary thyroid carcinoma, The Bethesda System for Reporting Thyroid Cytopathology

## Abstract

**Background:**

The Bethesda System for Reporting Thyroid Cytopathology (TBSRTC) predicts the risk of malignancy for the different categories of the ultrasound-guided fine-needle aspiration biopsy (FNAB). The objective of this study is to investigate the efficiencies of the v-raf murine sarcoma viral oncogene homolog B1 (*BRAF*) V600E mutation test and the TBSRTC categories in distinguishing between benign and malignant thyroid nodules.

**Methods:**

In this study, 362 ultrasound-guided fine-needle aspiration (FNA) samples from 344 patients aged from 17 to 76 years old were retrospectively reviewed. The patients were classified into six groups (I–VI) according to the TBSRTC system. The amplification refractory mutation system-polymerase chain reaction (ARMS-PCR) was used to evaluate the *BRAF* V600E mutation level in total 362 samples. Among of the 344 patients, 128 patients (131 thyroid nodules) who underwent surgeries were followed by histopathological examination. The predictive values of the *BRAF* V600E mutation test and TBSRTC categories were evaluated in these 131 thyroid nodules.

**Results:**

The median ages of the patients in the TBSRTC IV–VI group were smaller than those in the TBSRTC I–III groups. The proportion of nodules over 1 cm was larger than it in the TBSRTC IV group compared to the other groups. Significant differences in *BRAF* V600E mutation were observed (*P* < .001) among these six groups. The sensitivity (89.57%) for the detection of malignant thyroid nodules, negative predictive value (NPV; 45.45%) for the detection of benign nodules, and accuracy (86.26%) for distinguishing between benign and malignant thyroid nodules increased by combining the *BRAF* V600E mutation test and TBSRTC system when compared with the *BRAF* V600E mutation test and TBSRTC system respectively. The *BRAF* V600E mutation test alone demonstrated the increased positive predictive value (PPV; 98.91%) and specificity (93.75%) for the detection of malignant thyroid nodules compared to the TBSRTC method (alone or in combination with the *BRAF* V600E method).

**Conclusion:**

In summary, significant differences in age, nodule diameter, and *BRAF* V600E mutation were noted among the six categories of the TBSRTC system. The combination of the *BRAF* V600E mutation test and TBSRTC system demonstrated increases in the NPV, sensitivity, and accuracy, while the *BRAF* V600E method proved superiority to the TBSRTC system with regard to the PPV and specificity.

## 1 Introduction

With the development of ultrasound technology, the identification of people with thyroid nodules has improved.^[1]^ Papillary thyroid carcinoma (PTC) is the most common malignant histological subtype of thyroid nodules,^[2]^ of which the incidence has considerably increased worldwide.^[3–5]^ The overall prognosis of PTC is good with a 10-year survival rate of >90%.^[6–8]^ However, the recurrence rate has been reported to range from 20% to 30% especially in the neck lymph nodes.^[9,10]^ The early diagnosis and management of PTC continued remaining a priority. The combination of ultrasound-guided fine-needle aspiration biopsy (FNAB) and cytological evaluation has been conventionally used for the diagnosis of all types of high-risk thyroid nodules because ultrasound alone was unable to precisely distinguish thyroid cancers from benign thyroid diseases.^[11]^

According to The Bethesda System for Reporting Thyroid Cytopathology (TBSRTC),^[12]^ thyroid nodules are classified into six diagnostic categories as follows:

1. Non-diagnostic (ND) or Unsatisfactory (UNS);
2. Benign;
3. Atypia of Undetermined Significance (AUS) or Follicular Lesion of Undetermined Significance (FLUS);
4. Follicular Neoplasm (FN) or Suspicious for a Follicular Neoplasm (SFN);
5. Suspicious for Malignancy (SFM)
6. Malignant.

Moreover, the risk of malignancy for the six categories are 5% to 10%, 0% to 3%, 6% to 18%,10% to 40%, 45% to 60%, and 94% to 96% respectively, when non-invasive follicular thyroid neoplasm with papillary-like nuclear features (NIFTP) is not considered as a malignancy and 5% to 10%,0% to 3%, ∼10% to 30%, 25% to 40%, 50% to 75%, and 97% to 99% respectively, when NIFTP is included among the “carcinomas”.^[12]^ However, approximately 5–20% of fine-needle aspiration biopsy (FNA) specimens are reported to give inconclusive results which are read as “indeterminate” or “suspicious malignancy”. FNA can fail to allow discrimination between benign and malignant lesions for several reasons. This failure is a diagnostic problem for doctors and causes significant anxiety and uncertainty for patients. Discordant FNA results can make partial or total thyroidectomy necessary for diagnosis, though only 17–51% of these operations are actually in medical need.

In addition, the use of molecular testing for AUS or FLUS and FN or SFN has been suggested.^[12]^ Some studies have reported the involvement of the *BRAF* V600E mutation in PTC.^[13]^ A point mutation of the *BRAF* V600E oncogene, which results in a change from valine to glutamate in codon V600E, has been reported in 30%–80% of the PTC patients.^[14]^ Furthermore, the most commonly reported molecular event in PTC is a mutation in the proto-oncogene *BRAF*, specifically, the V600E mutation, with an average rate of 49% according to a recent meta-analysis.^[15]^ It has been suggested that preoperative knowledge of the *BRAF* mutation status may help to guide the extent of the initial surgery.^[14]^ The combination use of the *BRAF* V600E mutation test and FNA cytology could increase the accuracy of the diagnosis of PTC if they applied different categorization of nodules.^[16,17]^

In this study, we compared the association between the clinicopathological characteristics (including age, gender, tumor size, *BRAF* V600E mutation status and the histological types) among the different TBSRTC categories.

## 2 Methods

### 2.1 Patients and samples

Thyroid FNA samples from 362 thyroid nodules were obtained from 344 patients at the Pathology Department of the Sir Run Run Shaw Hospital, Medical School, Zhejiang University, China. All patients who underwent *BRAF* V600E testing with FNA specimens from October 2015 to March 2017 were selected in this retrospective study. Biopsies were obtained from the left and right thyroid lobes of 18 patients. Furthermore,128 patients (131 thyroid nodules) among of 344 patients underwent operations from October 2015 to November 2018; both lobes of the thyroid were operated in three patients. This study was approved by the Ethics Committee of the Biomedical Research Center at the Sir Run Run Shaw Hospital.

### 2.2 Cytological analysis

The FNA samples were obtained under the guidance of ultrasound specialists, using fine needles (25G × 40 mm, 23G × 50 mm, 22G × 70 mm) which were made by Hakko Co, Ltd. (Chikuma-Shi, Nagano, Japan). Each aspirate was uniformly smeared on a slide and fixed with alcohol, followed by hematoxylin and eosin staining. The stained slides were reviewed for routine cytological assessment by experienced pathologists according to the TBSRTC^[12]^ classification.

### 2.3 Deoxyribonucleic acid (DNA) extraction

DNA was isolated from the FNA samples and extracted by the QIAGEN kit (QIAamp DNA FFPE Tissue Kit, Product of Germany) following the manufacturer's protocol. The concentrations and qualities of the harvested DNA were determined by the Smart Spec Plus Spectrophotometer (BioRad Life Science, USA).

### 2.4 BRAF V600E mutation analysis by ARMS-PCR

The amplification refractory mutation system-polymerase chain reaction (ARMS-PCR) detection method was applied to evaluate the pattern of *BRAF* V600E mutation. DNA (10–15 ng) was added into the AmoyDx kit (ADx-ARMS, Amoy Diagnostics Co. Ltd, Xiamen, China) according to the manufacturer's instructions. Then, the mixture was reacted on a Light Cycler 480 II or cobas z 480 (Roche apparatus) via ARMS-PCR based on the following steps: 95°C for 5 min, 15 annealing cycles at 95°C for 25 s, 64°C for 20 s, and 72°C for 20 s; finally, 31 extension cycles were performed at 93°C for 25 s, 60°C for 35 s, and 72°C for 20 s. According to the manual, the cycle threshold (Ct) cutoff value to judge the *BRAF* V600E mutation status was 28. When the Ct value of a certain well was less than 28, the sample was considered as positive for the *BRAF* V600E mutation, and when it was equal to or more than 28, the sample was negative for the mutation.

### 2.5 Histological diagnosis

All the surgical specimens underwent conventional histological assessment. The histological diagnoses of the specimens were established according to the 2017 World Health Organization (WHO) Classification of Tumors of Endocrine Organs.^[18]^

### 2.6 Statistical analysis

Data were analyzed by the statistical software SPSS 22.0. Analysis of Variance was used to determine the differences between six groups. The difference was considered statistically significant when the *P*-value was ≤.05. The sensitivity was categorized as true positive/(true positive+ false negative); specificity was categorized as true negative/(true negative+ false positive); PPV was categorized as true positive/(true positive+ false positive); and NPV was categorized as true negative/(true negative+ false negative).

## 3 Results

### 3.1 Correlation between TBSRTC analysis and clinicopathological characteristics

All the classifications and clinicopathological characteristics, including sex, age, nodule diameter, and BRAF V600E mutation level were examined (Table 1). A significant difference in age (*P* < .001) was observed from the TBSRTC IV–VI groups compared to the TBSRTC I–III groups. The proportion of nodules greater than 1 cm was more than it in the TBSRTC IV group when compared with the other groups (TBSRTC I–VI). Groups TBSRTC I and TBSRTC IV were negative to the BRAF V600E mutation; however, the data obtained from these two groups was limited which might affect the results. The TBSRTC IV group was excluded with the increase in the number of groups (TBSRTC I–VI), the proportion of *BRAF* mutation positive increased (Table 1).

**Table 1:** Correlations between the clinicopathological characteristics and The Bethesda System for Reporting Thyroid Cytopathology categories.

	I(ND/UNS)	II(Benign)	III(AUS/FLUS)	IV(FN/SFN)	V(SFM)	VI(Malignant)	*P* value
N (N/%)	6 (1.7%)	88 (24.3%)	163 (45%)	4 (1.1%)	78 (21.5%)	23 (6.4%)	NA
Median age (Y)	53	51	49	39	40	39	<.001
Gender (N/%)							.337
Male	2 (33.3%)	20 (22.7%)	37 (22.7%)	1 (25%)	10 (12.8%)	7 (30.4%)	
Female	4 (66.7%)	68 (77.3%)	126 (77.3%)	3 (75%)	68 (87.2%)	16 (69.6%)	
Nodule diameter (N/%)							.041
Size ≤ 1.0cm	5 (83.3%)	57 (64.8%)	114 (69.8%)	1 (25%)	65 (83.3%)	16 (69.6%)	
Size > 1.0cm	1 (16.7%)	31 (35.2%)	49 (30.2%)	3 (75%)	13 (16.7%)	7 (30.4%)	
BRAF V600E mutation (N/%)							.000
Positive	0 (0%)	3 (3.4%)	44 (27%)	0 (0%)	51 (66.4%)	19 (82.6%)	
Negative	6 (100%)	85 (96.6%)	119 (73%)	4 (100%)	27 (34.6%)	4 (17.4%)	
Treatment (N)							NA
Observation or lost follow-up	5	77	117	4	22	6	
Surgery	1	11	46	0	56	17	
Histology							
Benign (N)	0	6	5	0	4	0	
Hashimoto's thyroiditis	0	1	1	0	2	0	
Tissue fibrosis, focal atypia	0	0	1	0	0	0	
Nodular goiter	0	3	1	0	2	0	
Adenomatoid nodules	0	2	2	0	0	0	
Hyalinizing trabecular tumor^∗^	0	0	0	0	1	0	
Malignant (N)	1 (16.7%)	5 (5.7%)	41 (25.15%)	0	51 (66.4%)	17 (73.9%)	
PTC	1	5	40	0	48	16	
mPTC	0	0	1	0	2	1	
Poorly differentiated Carcinoma	0	0	0	0	1	0	

ND = Non-diagnostic, UNS = Unsatisfactory, AUS = Atypia of Undetermined Significance, FLUS = Follicular Lesion of Undetermined Significance, FN = Follicular Neoplasm, SFN = Suspicious for a Follicular Neoplasm, SFM = Suspicious for Malignancy, PTC = Papillary thyroid carcinoma, mPTC = Papillary thyroid microcarcinoma, NA = Not applicable

∗ Hyalinizing trabecular tumor is a borderline tumor according to the 2017 World Health Organization Classification of Tumors of Endocrine Organs. We classified it as a benign tumor for the present discussion.

### 3.2 Correlation between BRAF V600E mutation and TBSRTC groups

The major histologic types of nodules observed were PTC (In this study, all PTC is papillary carcinoma, with papillary/non folllicular architecture.), papillary thyroid microcarcinoma (mPTC), poorly differentiated carcinoma, Hashimoto's thyroiditis, nodular goiter, adenomatoid nodules, and hyalinizing trabecular tumor. Thirty nine nodules among of 131 were negative for the *BRAF* V600E mutation; among of which, 24 were diagnosed with PTC, while the remaining 15 were benign lesions (Table 2). Alternatively, 92 cases were positive for the *BRAF* V600E mutation, including 88 (95.65%) were diagnosed as PTC and 3(3.26%) were mPTC after the surgery. No other histopathological type of malignant tumor was found. The remaining case was diagnosed with benign focal atypical fibrosis (Table 3). *BRAF* V600E mutation (close to the cutoff value) was observed in the DNA extracted from atypical regional tissue which was scraped from the formalin-fixed, paraffin-embedded (FFPE) surgical sample and diagnosed benign focal atypical fibrosis. The *BRAF* V600E mutation rate among the samples diagnosed with PTC and mPTC patients was 79.8% (91/114).

**Table 2:** Characteristics of the 39 nodules with negative BRAFV600E mutations.

	I(ND/UNS)	II(Benign)	III(AUS/FLUS)	IV(FN/SFN)	V(SFM)	VI(Malignant)
Ct = 0 (N)	0	9	11	0	14	2
Histology						
Benign (N)	/	6	3	/	5	0
Hashimoto's thyroiditis	/	1	1	/	2	0
Nodular goiter	/	3	2	/	2	0
Adenomatoid nodules	/	2	0	/	0	0
Hyalinizing trabecular tumor^∗^	/	0	0	/	1	0
Malignant (N)	/	3	8	/	9	2
PTC	/	3	8	/	8	2
Poorly differentiated carcinoma	/	0	0	/	1	0
28 ≤ Ct ≤ 31(N)	1	0	1	0	1	0
Histology						
Nodular goiter	0	/	1	/	0	/
PTC	1	/	0	/	1	/
Total	1	9	12	0	15	2

ND = Non-diagnostic, UNS = Unsatisfactory, AUS = Atypia of Undetermined Significance, FLUS = Follicular Lesion of Undetermined Significance, FN = Follicular Neoplasm, SFN = Suspicious for a Follicular Neoplasm, SFM = Suspicious for Malignancy, Ct = cycle threshold, PTC = Papillary thyroid carcinoma

∗ Hyalinizing trabecular tumor is a borderline tumor according to the 2017 World Health Organization Classification of Tumors of Endocrine Organs. We classified it as a benign tumor for the present discussion.

**Table 3:** Characteristics of the 92 nodules with positive BRAFV600E mutations.

	I(ND/UNS)	II(Benign)	III(AUS/FLUS)	IV(FN/SFN)	V(SFM)	VI(Malignant)
N	0	2	34	0	41	15
Histology						
PTC	/	2	32	0	40	14
mPTC	/	0	1	0	1	1
Focally atypical with fibrosis	/	0	1	0	0	0

ND = Non-diagnostic, UNS = Unsatisfactory, AUS = Atypia of Undetermined Significance, FLUS = Follicular Lesion of Undetermined Significance, FN = Follicular Neoplasm, SFN = Suspicious for a Follicular Neoplasm, SFM = Suspicious for Malignancy, PTC = Papillary thyroid carcinoma, mPTC = papillary thyroid microcarcinoma.

Associations among the TBSRTC categories, *BRAF* V600E mutations, and final diagnosis in the 131 nodules are shown in Table 4. The sensitivity (89.57%) for the detection of malignant thyroid nodules, negative predictive value (NPV; 45.45%) for the detection of benign nodules, and accuracy (86.26%) for distinguishing between benign and malignant thyroid nodules increased in the combination of the *BRAF* V600E mutation test and TBSRTC system when compared to the *BRAF* V600E mutation test and TBSRTC system, respectively. The *BRAF* V600E mutation test alone demonstrated the increased positive predictive value (PPV; 98.91%) and specificity (93.75%) for the detection of malignant thyroid nodules compared to the TBSRTC method (alone or in combination with the *BRAF* V600E method). (alone or in combination with the *BRAF* V600E method; Table 5).

**Table 4:** Associations between the Bethesda System for Reporting Thyroid Cytopathology categories, BRAF V600E mutation, and the final diagnosis for the 131 nodules examined.

	BRAFV600E		TBSRTC						Both		
Histopathology	Negative	Positive	I	II	III	IV	V	VI	Benign	Malignancy	Total
Benign	15	1	0	6	5	0	5	0	10	6	16
Malignant	24	91	1	5	41	0	51	17	12	103	115
Total	39	92	1	11	46	0	56	17	22	109	131

TBSRTC = The Bethesda System for Reporting Thyroid Cytopathology, TBSRTC = The Bethesda System for Reporting Thyroid Cytopathology.

**Table 5:** Comparison of the diagnostic values of the Bethesda System for Reporting Thyroid Cytopathology system, BRAF V600E mutation, and the combination of the two methods.

Method	PPV	NPV	Specificity	Sensitivity	Accuracy
TBSRTC	93.15%	18.97%	68.75%	59.13%	60.31%
BRAFV600E	98.91%	38.46%	93.75%	79.13%	80.92%
TBSRTC+BRAFV600E	94.50%	45.45%	62.50%	89.57%	86.26%

PPV = Positive predictive value, NPV = Negative predictive value, TBSRTC = The Bethesda System for Reporting Thyroid Cytopathology.

## 4 Discussion

PTC is the most common histological category of thyroid malignancy.^[2]^ It is well-known for its usually mild clinical course among the cancers with a very high 10-year survival rate (>90%).^[6–8]^ However, the recurrence rate has been reported to range between 20% and 30% especially in the neck lymph nodes.^[9,10]^ Thus, the early diagnosis of PTC is vital. The use of the traditional FNA cytology examination method alone has been proven to be unsuccessful in the identification of patients with PTC patients in a timely manner. Alternatively, *BRAF* V600E mutations have been reported to be a potential biomarker of the *BRAF* gene and may prove useful for identifying patients with PTC.^[19–21]^

The FNA sample categorization in the study is different from its in previous studies.^[16,17]^ In one study the nodules were classified as benign, follicular lesions (including follicular lesions of undetermined significance and follicular neoplasm/suspicious for follicular neoplasm), suspicious for malignancy, malignant and non-diagnostic, following the guidelines of National Cancer Institute thyroid fine needle aspiration (FNA) state of the science conference.^[16]^ According to The Bethesda System for Reporting Thyroid Cytopathology (TBSRTC), Thyroid nodules are classified into six diagnostic categories in this study according to The Bethesda System for Reporting Thyroid Cytopathology (TBSRTC) as mentioned in introduction where the follicular lesions category was divided into TBSRTC III (AUS/FLUS) and TBSRTC IV (FN/SFN) categories in their study.^[16]^

In another study the nodules were classified into four categories: malignant, indeterminate (including suspicious for malignancy and follicular neoplasm or lesion), benign, and inadequate.^[17]^ The indeterminate nodules in their study^[17]^ were divided into TBSRTC IV (FN/SFN) and TBSRTC V (SFM) categories in our study.

Samples of FL was included in their study^[16]^ and most samples (88/89) were *BRAF* V600E mutation negative while there were no samples of FN in this study. We detected *BRAF* V600E mutation by ARMS-PCR which is time-saving and more sensitive while *BRAF* V600E mutation is analyzed by direct sequencing in the former studies and RFLP^[16]^ or the DPO-based multiplex PCR which maybe produce false-positive results.^[17]^

We compared the different clinicopathological characteristics, including age, gender, tumor size, *BRAF* V600E mutations, and histological types among the six diagnostic categories proposed by TBSRTC in this study. In addition, the diagnostic values of the *BRAF* V600E mutation test and thyroid FNA cytology alone and in combination the *BRAF* V600E mutation test and thyroid FNA cytology in distinguishing benign from malignant thyroid nodules were evaluated. The combination of the *BRAF* V600E mutation test and TBSRTC system significantly increased the sensitivity, NPV, and accuracy compared to the *BRAF* V600E mutation test or TBSRTC system respectively (Table 5) as former result.^[16]^ Furthermore, the sensitivity of the *BRAF* V600E mutation rate was 79.8% (91/114). Interestingly, one nodule with *BRAF* V600E mutation was diagnosed as TBSRTCIII, while the surgical specimen obtained from this patient indicated a diagnosis of focal atypical fibrosis; in addition, the specimen presented with *BRAF* V600E mutation (close to the cutoff value). A study has reported the detection of *BRAF* mutations in benign thyroid tissues.^[22]^ This may be attributed to the unique genetic changes within tumors. There may be a loss of evidence of morphological changes in the tissue during the extended period from DNA duplication and transcription to translation. Additional studies are required to evaluate this theory.

Samples from three patients demonstrated Ct values higher than 28, but lower than 30, and two of the three patients were diagnosed with PTC. One sample belonged to the TBSRTC I (ND/UNS) group, while the other belonged to the TBSRTC V (SFM) group. The number of cells from these two samples might be too low to obtain accurate results. Another patient who presented with late amplification was diagnosed with nodular goiter and atypical follicular epithelium, which corresponded with the cytopathological diagnosis of AUS/FLUS.

It should be noted that this study has its limitations since only 344 patients were investigated, and the quantity of sample of patients with ND and FN were particularly small. The pathological diagnosis in some patients were not confirmed, whereas some patients were lost to follow-up. The patients selected for the surgical procedures were mainly diagnosed with malignant tumors. Therefore the study results may be biased and more study samples are required to validate these findings.

## 5 Conclusion

This study has shown that the combination of the TBSRTC method and *BRAF* V600E mutation examination can improve the sensitivity, NPV, and accuracy for the identification of patients with PTC or mPTC when compared with the *BRAF* V600E mutation test or TBSRTC system respectively. Moreover, the *BRAF* V600E mutation test may be used to distinguish between benign and malignant thyroid nodules (Malignant thyroid nodules is mainly referring to PTC and mPTC in our study.), based on the findings of the present study. This mutation is a useful molecular marker and should be routinely tested. The traditional approach involves an increase in the frequency of follow-ups for these suspicious patients. And they may do once more FNA which is traumatic in order to diagnose. Currently, *BRAF* V600E co-testing in thyroid FNA cytology has proven to be economical and convenient by reducing the number of follow-up visits for the patient; in addition, it has an auxiliary diagnostic significance for the doctors.

## Acknowledgments

We thank Dr. Xu Sung-Xiao (Clinical Lab, Zhejiang Cancer Hospital, Hangzhou, China) for helping in the paper.

## Author contributions

**Conceptualization:** Danli Sheng.

**Data curation:** Danli Sheng.

**Formal analysis:** Danli Sheng.

**Funding acquisition:** Jianzhong Chen.

**Investigation:** Danli Sheng, Xiaoli Yu, Hui Li.

**Methodology:** Danli Sheng, Xiaoli Yu, Hui Li, Murui Zhang.

**Project administration:** Jianzhong Chen.

**Resources:** Jianzhong Chen.

**Software:** Xiaoli Yu, Hui Li.

**Supervision:** Jianzhong Chen.

**Validation:** Hui Li, Jianzhong Chen.

**Visualization:** Hui Li, Jianzhong Chen.

**Writing – original draft:** Danli Sheng, Xiaoli Yu, Hui Li, Murui Zhang.

**Writing – review & editing:** Danli Sheng, Xiaoli Yu, Jianzhong Chen.

## References

[1] Bhartiya R Mallik M Kumari N Prasad BN. Evaluation of thyroid lesions by fine-needle aspiration cytology based on Bethesda system for reporting thyroid cytopathology classification among the population of South Bihar. Indian J Med Paediatr Oncol Off J Indian Soc Med Paediatr Oncol 2016;37:265–70. 10.4103/0971-5851.195742 PMC5234164 28144094

[2] Rosenbaum MA McHenry CR. Contemporary management of papillary carcinoma of the thyroid gland. Expert Rev Anticancer Ther 2009;9:317–29. 19275510 10.1586/14737140.9.3.317

[3] Davies L Welch HG. Increasing incidence of thyroid cancer in the United States, 1973–2002. JAMA 2006;295:2164–7. 16684987 10.1001/jama.295.18.2164

[4] Wang Y Wang W. Increasing incidence of thyroid cancer in Shanghai, China, 1983–2007. Asia-Pac J Public Health 2015;27:N223–9. 10.1177/1010539512436874 22345304

[5] Netea-Maier RT Aben KK Casparie MK. Trends in incidence and mortality of thyroid carcinoma in the Netherlands between 1989 and 2003: correlation with thyroid fine-needle aspiration cytology and thyroid surgery. Int J Cancer 2008;123:1681–4. 18623130 10.1002/ijc.23678

[6] Sciuto R Romano L Rea S. Natural history and clinical outcome of differentiated thyroid carcinoma: a retrospective analysis of 1503 patients treated at a single institution. Ann Oncol 2009;20:1728–35. 19773250 10.1093/annonc/mdp050

[7] Toniato A Boschin I Casara D Mazzarotto R Rubello D Pelizzo M. Papillary thyroid carcinoma: factors influencing recurrence and survival. Ann Surg Oncol 2008;15:1518–22. 18324441 10.1245/s10434-008-9859-4

[8] Collet JF Lacave R Hugonin S Poulot V Tassart M Fajac A. BRAF V600E detection in cytological thyroid samples: a key component of the decision tree for surgical treatment of papillary thyroid carcinoma. Head Neck 2016;38:1017–21. 26854757 10.1002/hed.24393

[9] Simon D Goretzki PE Witte J Röher HD. Incidence of regional recurrence guiding radicality in differentiated thyroid carcinoma. World J Surg 1996;20:860–6. discussion 866. 8678963 10.1007/s002689900131

[10] Hay ID McConahey WM Goellner JR. Managing patients with papillary thyroid carcinoma: insights gained from the Mayo Clinic's experience of treating 2,512 consecutive patients during 1940 through 2000. Trans Am Clin Climatol Assoc 2002;113:241–60. 12053713 PMC2194488

[11] Haugen BR Alexander EK Bible KC. American Thyroid Association management guidelines for adult patients with thyroid nodules and differentiated thyroid cancer: the American Thyroid Association Guidelines Task Force on Thyroid Nodules and Differentiated Thyroid Cancer. Thyroid 2016;26:01–133. 10.1089/thy.2015.0020 PMC4739132 26462967

[12] Cibas ES Ali SZ. The 2017 Bethesda system for reporting thyroid cytopathology. J Am Soc Cytopathol 2017;6:217–22. 31043290 10.1016/j.jasc.2017.09.002

[13] Xing M. BRAF mutation in thyroid cancer. Endocr Relat Cancer 2005;12:245–62. 15947100 10.1677/erc.1.0978

[14] Yip L Nikiforova MN Carty SE. Optimizing surgical treatment of papillary thyroid carcinoma associated with BRAF mutation. Surgery 2009;146:1215–23. 19958951 10.1016/j.surg.2009.09.011

[15] Lee JH Lee ES Kim YS. Clinicopathologic significance of BRAF V600E mutation in papillary carcinomas of the thyroid: a meta-analysis. Cancer 2007;110:38–46. 17520704 10.1002/cncr.22754

[16] Zatelli MC Trasforini G Leoni S. BRAF V600E mutation analysis increases diagnostic accuracy for papillary thyroid carcinoma in fine-needle aspiration biopsies. Eur J Endocrinol 2009;161:467–73. 19574281 10.1530/EJE-09-0353

[17] Kim SW Lee JI Kim JW. BRAFV600E mutation analysis in fine-needle aspiration cytology specimens for evaluation of thyroid nodule: a large series in a BRAFV600E-prevalent population. J Clin Endocrinol Metab 2010;95:3693–700. 20501689 10.1210/jc.2009-2795

[18] WHO Classification of Tumors of Endocrine Organs edited by, Lloyd RV, Osamura RY, Öppel GKl,Rosal J. International Agency for Research on Cancer and Lyon 2017.

[19] Xing M Tufano RP Tufaro AP. Detection of BRAF mutation on fine needle aspiration biopsy specimens: a new diagnostic tool for papillary thyroid cancer. J Clin Endocrinol Metab 2004;89:2867–72. 15181070 10.1210/jc.2003-032050

[20] Elisei R Ugolini C Viola D. BRAF(V600E) mutation and outcome of patients with papillary thyroid carcinoma: a 15-year median follow-up study. J Clin Endocrinol Metab 2008;93:3943–9. 18682506 10.1210/jc.2008-0607

[21] Xing M Alzahrani AS Carson KA. Association between BRAF V600E mutation and mortality in patients with papillary thyroid cancer. JAMA 2013;309:1493–501. 23571588 10.1001/jama.2013.3190 PMC3791140

[22] Jara SM Bhatnagar R Guan H. Utility of BRAF mutation detection in fine-needle aspiration biopsy samples read as “suspicious for papillary thyroid carcinoma”. Head Neck 2015;37:1788–93. 24989827 10.1002/hed.23829

